# Genetic Diversity, Population Structure, and Linkage Disequilibrium of a Core Collection of *Ziziphus jujuba* Assessed with Genome-wide SNPs Developed by Genotyping-by-sequencing and SSR Markers

**DOI:** 10.3389/fpls.2017.00575

**Published:** 2017-04-18

**Authors:** Wu Chen, Lu Hou, Zhiyong Zhang, Xiaoming Pang, Yingyue Li

**Affiliations:** ^1^National Engineering Laboratory for Tree Breeding, College of Biological Sciences and Technology, Beijing Forestry UniversityBeijing, China; ^2^Beijing Key Laboratory of Ornamental Plants Germplasm Innovation and Molecular Breeding, National Engineering Research Center for Floriculture, Beijing Laboratory of Urban and Rural Ecological Environment, Key Laboratory of Genetics and Breeding in Forest Trees and Ornamental Plants of Ministry of Education, School of Landscape Architecture, Beijing Forestry UniversityBeijing, China

**Keywords:** jujube core collection, genotyping-by-sequencing (GBS), SNPs, SSRs, genetic diversity, population structure, linkage disequilibrium (LD)

## Abstract

Chinese jujube (*Ziziphus jujuba* Mill) is an economically important fruit species native to China with high nutritious and medicinal value. Genotyping-by-sequencing was used to detect and genotype single nucleotide polymorphisms (SNPs) in a core collection of 150 Chinese jujube accessions and further to characterize their genetic diversity, population structure, and linkage disequilibrium (LD). A total of 4,680 high-quality SNPs were identified, of which 38 sets of tri-allelic SNPs were detected. The average polymorphism information content (PIC) values based on bi-allelic SNPs and tri-allelic SNPs were 0.27 and 0.38, respectively. STRUCTURE and principal coordinate analyses based on SNPs revealed that the 150 accessions could be clustered into two groups. However, neighbor-joining trees indicated the accessions should be grouped into three major clusters. Our data confirm that the resolving power for genetic diversity was similar for the SSRs and SNPs. In contrast, regarding population structure, the resolving power was higher for SSRs than for SNPs. The LD pattern in Chinese jujube was investigated for the first time. We observed a relatively rapid LD decay with a short range (∼10 kb) for all pseudo-chromosomes and for individual pseudo-chromosomes. Our findings provide important information for future genome-wide association analyses and marker-assisted selective breeding of Chinese jujube.

## Introduction

Chinese jujube (*Ziziphus jujuba* Mill) is a diploid species (2*n* = 2*x* = 24; genome size: 437.65 Mb), and is one of the most important fruit tree species native to China (Liu and Wang, 2009; Liu M. et al., 2014). Jujube fruits are highly nutritious with medicinal properties. They are an excellent source of vitamin C, phenolic compounds, carbohydrates and minerals (particularly potassium and iron), and cyclic AMP (Cyong and Hanabusa, 1980; Li et al., 2007; Gao et al., 2013). Chinese jujube plants are distributed throughout China, with the exception of Heilongjiang province, and have been introduced to more than 30 countries so far (Wang et al., 2014).

Information regarding genetic diversity and population structure is important for characterizing the domestication history and genetic relationships of Chinese jujube accessions. It may also be useful for accelerating the development of highly efficient breeding strategies. Many studies focused on Chinese jujube genetic diversity and population structure have been conducted because of advances in molecular marker techniques, including the application of random amplified polymorphic DNA markers (Liu et al., 2005), amplified fragment length polymorphisms (Bai, 2008; Qiao et al., 2009), sequence-related amplified polymorphisms (Bai, 2008), and simple sequence repeats (SSRs) (Ma et al., 2011; Wang et al., 2014; Xiao et al., 2015). However, all of these previous studies involved fewer than 100 cultivars. Twenty-four SSR markers were recently used to investigate 962 jujube accessions. The genetic diversity and population structure of these accessions were estimated and a core collection of 150 accessions was selected (Xu et al., 2016). The non-random association of alleles at two or more loci [i.e., linkage disequilibrium (LD)] is crucial for plant breeding. LD has been investigated in fruit species such as sweet cherry (Campoy et al., 2016), apple (Kumar et al., 2012), and grape (Lijavetzky et al., 2007). The distance over which an LD persists determines the number and density of markers, and affects how association analyses are conducted (Flint-Garcia et al., 2003). However, there is currently no available information regarding LD in Chinese jujube.

Among the available molecular markers, SSR (i.e., microsatellite DNA) and SNP (i.e., single nucleotide polymorphism) markers are useful for investigations of plant population genetics (Hamblin et al., 2007; Würschum et al., 2013; Filippi et al., 2015). Given the increasing use of SSR and SNP markers, a comparison of their utility in analyses of genetic diversity and population structure is warranted. Yang et al. (2011), Emanuelli et al. (2013), and Filippi et al. (2015) reported that the resolving power regarding genetic diversity was lower for SNPs than for SSRs, which contradicted the conclusions of Singh et al. (2013). In terms of population structure, Singh et al. (2013) and Müller et al. (2015) determined that the resolving power was higher for SNP markers than for SSR markers, while Hamblin et al. (2007) and Yang et al. (2011) reported the opposite conclusion. Nevertheless, Filippi et al. (2015) concluded that SSR and SNP markers produce similar results under a Bayesian approach.

The results of large scale studies revealed that genotyping-by-sequencing (GBS) is useful for identifying high density SNP markers and genotypes (Rocher et al., 2015; Torkamaneh and Belzile, 2015). It is also relevant for genetic diversity and population structure analyses (Burrell et al., 2015; Kujur et al., 2015; Sehgal et al., 2015). GBS technology was recently used to develop SNP markers, which were then used to construct a jujube genetic map (Zhang et al., 2016). A similar study was conducted based on restriction-site associated DNA technology (Zhao et al., 2014). However, there are no reports describing the development of SNP markers that were subsequently applied for analyzing genetic diversity and population structure in several jujube cultivars.

In the present study, we used GBS technology to study a core collection of 150 Chinese jujube accessions. Our objectives were to (1) detect and genotype SNPs at a genome-wide scale, (2) compare the performance of SSR and SNP markers by estimating the genetic diversity and population structure, and (3) characterize the LD pattern. The data presented herein may be useful for selecting appropriate SSRs and SNPs for different types of jujube analyses. Additionally, our findings may facilitate future genome-wide association studies and marker-assisted selective breeding of Chinese jujube.

## Materials and Methods

### Plant Materials

We used a core collection of 150 Chinese jujube accessions previously characterized by Xu et al. (2016) (Supplementary Table S1). Accessions were collected all over China and were planted at the following two locations using standard cultivation conditions: the National Chinese Jujube Germplasm Repository located in Taigu County, Shanxi Province, China and the National Foundation for Improved Cultivar of Chinese Jujube, Cangzhou County, Hebei Province, China. Fresh healthy leaves for each accession were collected (with permission) and then were immediately frozen in liquid nitrogen and stored at -80°C until used.

### DNA Extraction

Total genomic DNA was extracted from fresh leaves using plant genomic DNA rapid extraction kit developed by Biomed Gene Technology Co., Ltd., Beijing, China. The integrity, purity, and concentration of the extracted DNA were determined by 1% agarose gel electrophoresis and a Qubit Fluorometer (Invitrogen).

### Genotyping-by-sequencing Library Preparation and Sequencing

We digested 100 ng genomic DNA with *ApeK*I. The resulting samples were ligated to common and barcode adapters. The ligated products were pooled in equal volumes and then purified with the QIAquick PCR Purification Kit (Qiagen). Polymerase chain reaction (PCR) amplifications were conducted using the PCR Primer Cocktail and PCR Master Mix to enrich the adapter-ligated DNA fragments. The amplicons corresponding to target fragments were purified using the QIAquick Gel Extraction Kit (Qiagen) following 2% agarose gel electrophoresis. The average DNA fragment length for the final library was determined using the Agilent DNA 12,000 kit and 2100 Bioanalyzer system (Agilent). Quantitative real-time PCR with a TaqMan probe was used to quantify the final library. We amplified specific DNA fragments (180–480 bp) on the cBot instrument to generate DNA clusters on a flowcell, after which 100-bp paired-end sequencing was completed using the HiSeq 4000.

### Sequence Data Analysis and SNP Genotyping

Raw Illumina DNA sequence reads were de-multiplexed according to the barcodes, and the adapter/barcode sequences were trimmed using a custom C script. Reads in which more than half of the bases had quality values ≤5 and those able to be mapped to multiple locations were discarded. The generated clean data were then aligned against the jujube reference genome sequence (Assembly version: ZizJuj_1.1; Liu M. et al., 2014) using the Burrows–Wheeler Aligner (version 0.7.10) with default parameters (Li and Durbin, 2009). The variants were called using the GATK program (version 3.2.2) (McKenna et al., 2010) with parameter settings below: Quality by depth < 2.0, mapping quality < 40.0, read position rank sum test < -8.0, Fisher strand > 60.0, haplotype score > 13.0, mapping quality rank sum test < -12.5, while the genotype was called ultimately using a custom perl script [filter conditions: missing data < 20%; minor allele frequency (MAF) > 0.05].

### SSR Genotyping

A set of 24 SSRs distributed throughout the jujube genome was used to genotype all 150 Chinese jujube accessions. Details regarding the genotyping were previously described (Xu et al., 2016).

### Analysis of Genetic Diversity

The number of alleles and allele frequencies for SNP data (bi-allelic and tri-allelic SNPs) were calculated using the VCFtools program (Danecek et al., 2011). For SSR data, these parameters were calculated using the GenAlEx program (version 6.5) (Peakall and Smouse, 2012). The polymorphism information content (PIC) values for the SSR and SNP data were calculated using the following equation (Botstein et al., 1980):

$$PIC=1-\overset{n}{\underset{i=1}{\Sigma }}P_{i}^{2}-\overset{n-1}{\underset{i=1}{\Sigma }}\overset{n}{\underset{j=i+1}{\Sigma }}{2P}_{i}^{2}P_{j}^{2}$$

### Analysis of Population Structure

For the SSR genotyping data, a Bayesian clustering analysis was implemented in the STRUCTURE program (version 2.3.3) (Pritchard et al., 2000; Falush et al., 2003) to evaluate the population genetic structure. An admixture model and correlated allele frequencies were applied to estimate the ancestry fractions of each cluster attributed to each accession. For each K-value (range: 1–8), 20 independent runs were completed with a burn-in period of 100,000, followed by 100,000 Markov chain Monte Carlo repetitions. Parameters were set to default values, and all accessions were considered to have unknown origins. The delta-K method was implemented in the Structure Harvester program (Earl, 2012) to determine the most probable K-value. Accessions with membership probabilities ≥0.50 were considered to belong to the same group. An unrooted neighbor-joining phylogenetic tree (Nei’s genetic distance; 1,000 bootstrap replicates) was constructed using the PowerMarker program (version 3.51) (Liu and Muse, 2004).

For the genotyping data of 4,680 high-quality SNPs (MAF ≥ 0.05; missing data: <20%), we used the method described by Evanno et al. (2005) to determine the delta-K value. Briefly, we plotted the mean likelihood [L(K)] value over 20 runs for each K-value (range: 1–8). We estimated delta-K using the following formula: ΔK = m(|L,(K)|)/s[L(K)] (Evanno et al., 2005). The population genetic structure was determined using the Frappe program according to the delta-K value (Tang et al., 2005). An unrooted neighbor-joining phylogenetic tree was constructed using the MEGA program (version 6.0) based on the distance matrix, with 1,000 bootstrap replicates (Tamura et al., 2013).

Genetic distances between pairs of accessions were calculated and a principal coordinate analysis (PCoA) was completed for the SSRs and SNPs using the GenAlEx program (version 6.5) (Peakall and Smouse, 2012).

### Estimation of Linkage Disequilibrium

The pairwise LD between 4,680 genome-wide SNPs for all pseudo-chromosomes and individual pseudo-chromosomes in 150 Chinese jujube accessions was calculated based on the allele frequency correlations (*r*^2^) using the PopLDdecay program^1^. The LD decay was calculated when the *r*^2^ value decreased below a threshold level (i.e., *r*^2^ < 0.1). Mean *r*^2^ values were used to calculate the LD using a 100-kb sliding window-based approach.

## Results

### Genome-wide SNPs Discovery and Genotype using a GBS Assay

A GBS assay of the sequencing of 96-plex *ApeK*I-digested libraries constructed from 150 Chinese jujube accessions was conducted using an Illumina HiSeq 4000. After the primary quality filtering step, 144.0 Gb clean reads were generated (2.3-fold sequencing depth), with an average of 0.99 Gb reads (range: 0.208–3.32 Gb reads) per accession. BJFU-435 was excluded because of a lack of sufficient clean data (Supplementary Table S2). Using reference genome sequences approach, SNPs were detected and genotyped by the GATK program (version 3.2.2) (McKenna et al., 2010). With a minimal set of initial quality filters, a total of 105, 106 SNPs were identified. Restricting the filter conditions to SNPs, the genotyping data considerably decreased the number of SNPs to 91,702 (data not shown). Furthermore, 4,680 high-quality SNPs were identified, including 38 sets of tri-allelic SNPs (Supplementary Table S3). Although there was an insufficient amount of clean data for BJFU-435, a genome-wide search identified 23 SNPs (Supplementary Tables S2, S3). The average heterozygosity rate for all SNPs was 10.48% (Supplementary Table S4). We determined that 74.94% of the SNPs had a quality value of 998, while the remaining 25.06% had an average quality value of 574 (Supplementary Table S5). These values confirmed the authenticity of the 4,680 SNPs.

Among the 4,680 high-quality SNPs, 4,005 (85.6%) were physically mapped across 12 jujube pseudo-chromosomes, with an average map density of 81.79 kb. A genome-wide SNP density plot revealed that most SNPs were physically mapped on jujube pseudo-chromosome 1 (12.29%, 575 SNPs). The average marker density was 81.79 kb. The highest and lowest marker densities were observed on pseudo-chromosome 7 (62.54 kb) and pseudo-chromosome 3 (99.91 kb), respectively. The remaining 675 SNPs were physically mapped on unanchored scaffolds of the jujube genome with a marker density of 169.27 kb (**Table 1**). Transitions (2,896 allelic sites, 61.38%) were more frequent than transversions (1,822 allelic sites, 38.62%), with a ratio of 1.59. The A/G transitions and G/C transversions occurred at the highest and lowest frequencies, respectively. The frequencies of the two types of transitions were similar (i.e., A/G 31.07% and C/T 30.31%), as were the frequencies of the four types of transversions (i.e., A/C 9.81%, A/T 9.81%, G/C 8.97%, and G/T 10.03%) (**Table 2**).

**Table 1:** Genomic distribution of 4,680 single nucleotide polymorphisms (SNPs) physically mapped on 12 jujube pseudo-chromosomes and unanchored scaffolds.

Pseudo-chromosomes	Size (Mb)of pseudo-chromosomes	Numbers of SNPs	Percentage of SNPs	Average density (kb)
1	41.01	575	12.29%	73.03
2	30.52	400	8.55%	78.13
3	29.56	303	6.47%	99.91
4	26.70	401	8.57%	68.19
5	26.70	274	5.85%	99.79
6	25.75	352	7.52%	74.91
7	24.80	406	8.68%	62.54
8	24.80	316	6.75%	80.35
9	22.89	269	5.75%	87.13
10	20.03	247	5.28%	83.03
11	20.03	253	5.41%	81.06
12	19.07	209	4.47%	93.45
Total	311.85	4,005	85.59%	NA
Average	NA	NA	NA	81.79
Unanchored scaffolds	111.58	675	14.42%	169.27


**Table 2:** Percentage of transition and transversion SNPs identified using genotyping-by-sequencing (GBS) assay.

	Transitions		Transversions			
		
	A/G	C/T	A/C	A/T	G/C	G/T
Numbers of allelic sites	1,466	1,430	463	463	423	473
Percentage of allelic sites	31.07%	30.31%	9.81%	9.81%	8.97%	10.03%
Total (Percentage)	2,896 (61.38%)		1,822 (38.62%)			


### Comparison of SSR and SNP Markers Related to Genetic Diversity

The genetic diversity of 150 Chinese jujube accessions was evaluated using 24 SSRs, 38 sets of tri-allelic SNPs, and 4,642 bi-allelic SNP markers. The PIC value, the number of alleles, and the allele frequency spectrum were calculated.

**FIGURE 1:**
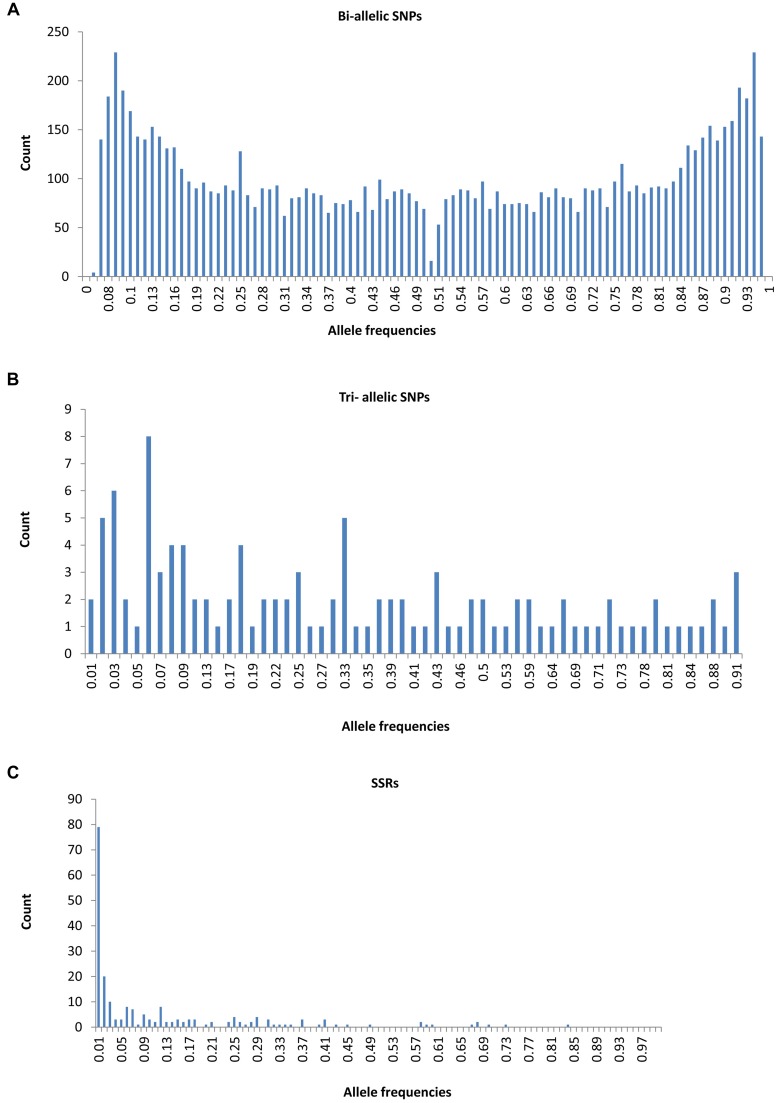
**Allele frequency spectra for different sets of markers in the 150 Chinese jujube accessions.** **(A)** 4,642 Bi-allelic single nucleotide polymorphisms (SNPs), **(B)** 38Tri- allelic SNPs, **(C)** 24 simple sequence repeats (SSRs).

A total of 209 alleles were generated from the 24 SSRs, with an average of 8.92 per locus. The highest average PIC value (0.59) was associated with the SSRs, followed by the tri-allelic SNPs (0.38), and the bi-allelic SNP markers (0.27) (**Table 3**). The allele frequency spectra were very different for the three data sets (**Figure 1**). We determined that 78.95% of the SSR alleles were present in the population at a low frequency (i.e., 0–0.2) (**Table 3** and **Figure 1C**). In contrast, 53.71% of the bi-allelic SNPs and 48.25% of the tri-allelic SNPs were present in the population at an intermediate frequency (i.e., 0.2–0.8) (**Table 3** and **Figures 1A,B**). Among the alleles present in the population at a high frequency (i.e., 0.8–1), the bi-allelic SNPs were the most abundant, followed by the tri-allelic SNPs and then the SSRs (**Table 3**).

**Table 3:** Genetic Diversity calculated by simple sequence repeats (SSRs), Bi- allelic SNPs, and Tri-allelic SNPs.

	Loci	PIC	Alleles	Average alleles per loci	Percentage of allele frequencies		
					
					0–0.2	0.2–0.8	0.8–1
SSRs	24	0.59	209	8.92	78.95%	20.57%	0.48%
Tri-allelic SNPs	38	0.38	114	3	42.98%	48.25%	8.77%
Bi- allelic SNPs	4,642	0.27	9,284	2	23.17%	53.71%	23.13%


The cost of the consumable laboratory supplies for the analyses of 150 jujube accessions using SSR markers and GBS-SNPs, as well as the cost per polymorphic locus are provided in **Table 4** and Supplementary Table S8 (all costs are in US dollars). The total cost of the GBS-SNP procedure was nearly four times that of the SSR procedure. However, the estimated cost of the genotyping supplies per polymorphic locus for the SSR procedure was $118.33. The corresponding estimated cost for the GBS-SNP procedure was $2.33. Therefore, the SSR procedure was about 51 times more expensive than the GBS-SNP procedure (per polymorphic locus).

**Table 4:** Consumable laboratory supplies costs in USD ($) for major steps in SSRs and GBS-SNPs procedure.

Step for SSRs	Cost for 150 accessions (US$)	Cost of per polymorphic locus (USD)	Step for GBS-SNPs	Cost for 150 accessions (USD)	Cost of per polymorphic locus (USD)
(1) DNA extraction	109.2	4.55	(1) DNA extraction	109.2	0.02
(2) Primers synthesis	143.38	5.97	(2) Digestion	1,638	0.35
(3) PCR	143.54	5.98	(3) Adapter ligated	1856.4	0.40
(4) Capillary electrophoresis	2443.88	101.83	(4) Pooling and purification	2184	0.47
			(5) PCR and purification	2511.6	0.54
			(6) Sequencing	2620.8	0.56

**Total**	2,840	118.33	**Total**	10,920	2.33


*Twenty-four polymorphic locus for SSR procedure and 4,680 polymorphic locus for GBS-SNPs procedure.*

### Comparison of SSR and SNP Markers Related to Population Structure

**FIGURE 2:**
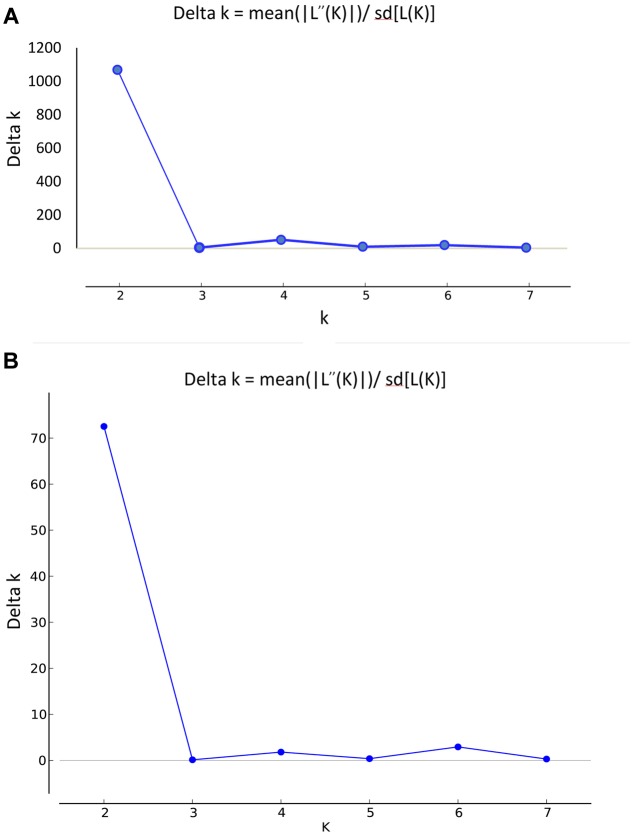
**Estimation of population using LnP(D) derived ΔK with K ranged from 1 to 8.** **(A)** 4,680 SNPs, **(B)** 24 SSRs.

**FIGURE 3:**
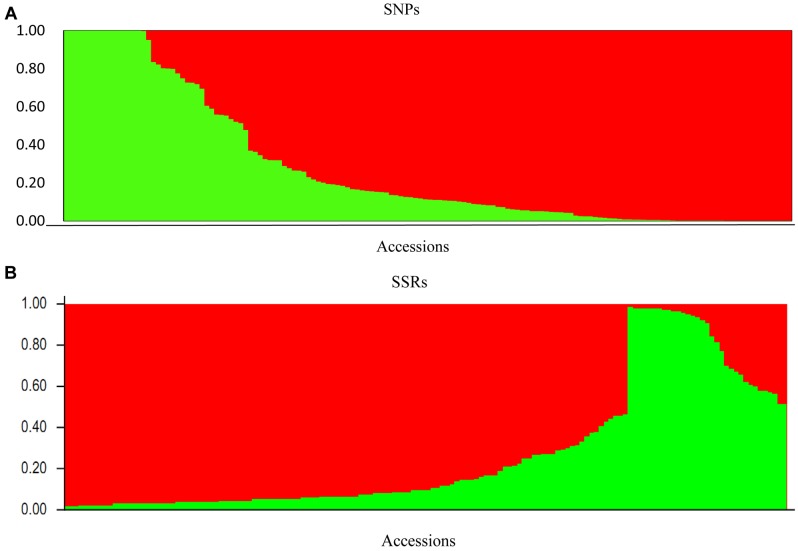
**Population structure (K = 2) inferred based on** **(A)** 4,680 SNPs, **(B)** 24 SSRs.

For the data sets of 4,680 SNPs and 24 SSRs, we observed a clear delta-K peak at K = 2 (**Figures 2A,B**) when the accessions were roughly divided into two major groups. Furthermore, based on the results of the STRUCTURE analysis, the accession with a score higher than 0.80 was considered to be a pure one, while it with a score lower than 0.80 was considered to be admixture one. For the SSRs, 33 accessions (19 pure and 14 admixture) were grouped into the green cluster and 117 accessions (90 pure and 27 admixture) were grouped into the red cluster (**Figure 3A**). For the SNPs, 37 accessions (23 pure and 14 admixture) were grouped into the green cluster, while 113 accessions (96 pure and 17 admixture) were grouped into the red cluster (**Figure 3B**). The classification of accessions into the two groups was consistent for the two marker types, with only 18 accessions classified into different groups (data not shown).

**FIGURE 4:**
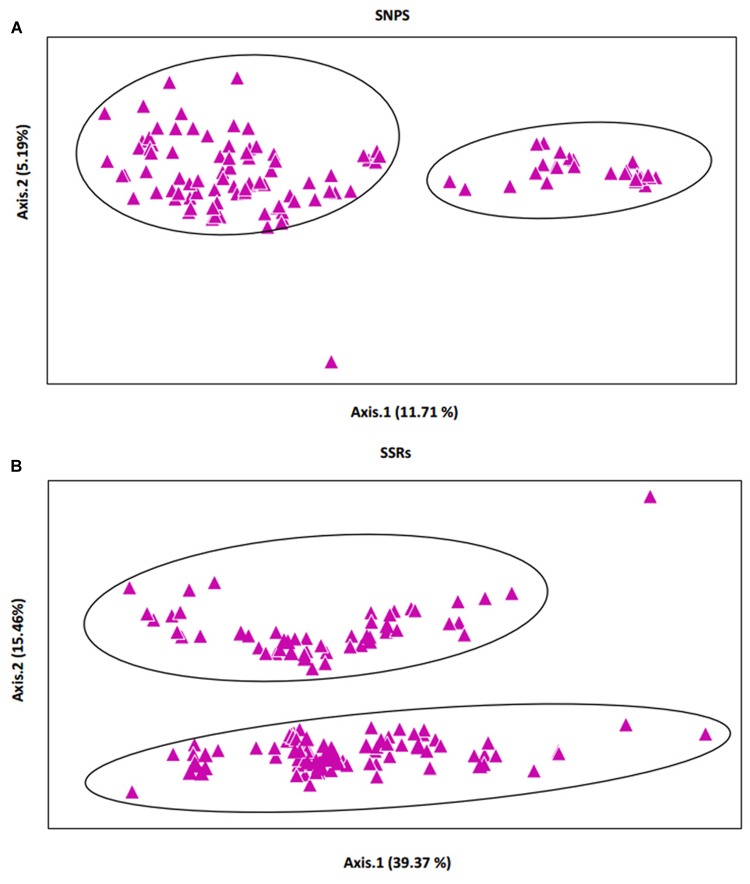
**Scatter plot from a principal coordinate analysis (PCoA).** PCoA of the 150 Chinese jujube accessions based on for the following sets of markers. **(A)** 4,680 SNPs, **(B)** 24 SSRs.

A PCoA based on the SSR and SNP markers revealed that the 150 Chinese jujube accessions were clearly separated into two broad groups across the first two axes (**Figures 4A,B**). The proportion of genotypic variance explained by the first two principal coordinates was higher for the SSRs (**Figure 4B**) than for the SNPs (**Figure 4A**). The first three SSR axes explained 63.06% of the cumulative variation, while the SNP datasets explained 20.83% of the variation (**Table 5**).

**Table 5:** Percentage of variation explained by the first three axes.

	SNPs			SSRs		
Axis	1	2	3	1	2	3
%	11.71	5.19	3.93	39.37	15.46	8.23
Cum %	11.71	16.90	20.83	39.37	54.82	63.06


**FIGURE 5:**
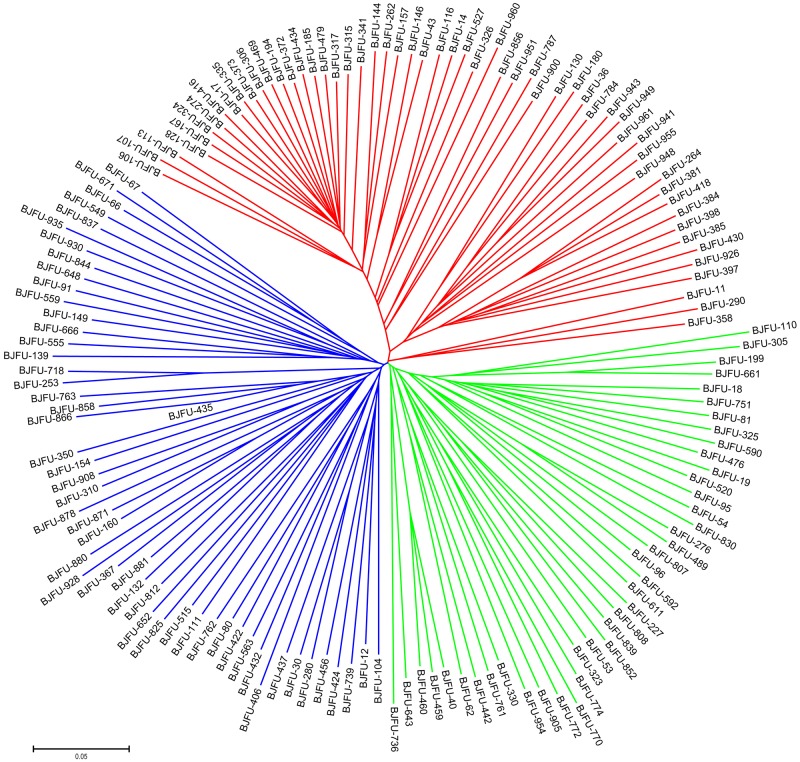
**Neighbor-joining dendrograms based on a simple matching dissimilarity matrix representing the grouping of the 150 Chinese jujube accessions for 4,680 SNPs**.

**FIGURE 6:**
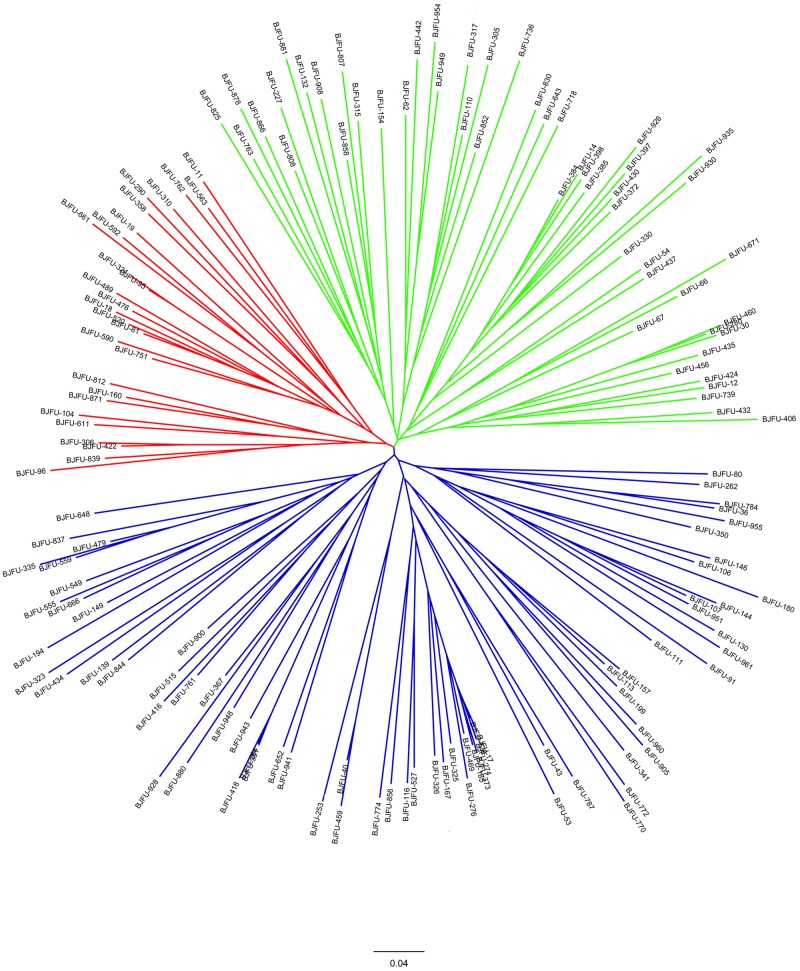
**Neighbor-joining dendrograms based on a simple matching dissimilarity matrix representing the grouping of the 150 Chinese jujube accessions for 24 SSRs**.

Neighbor-joining trees were constructed based on the SSR and SNP markers. In the tree constructed using SSR data, the 150 Chinese jujube accessions were grouped into three major clusters (**Figure 5**). The clusters labeled in green, blue, and red contained 51, 72, and 27 accessions, respectively. The jujube accessions were also grouped into three major clusters in the phylogenetic tree based on SNP data (**Figure 6**). The clusters labeled in green, blue, and red included 50, 59, and 41 accessions, respectively. In both trees, 23, 41, and 15 accessions were consistently classified into the green, blue, and red clusters, respectively. These clustering results provide evidence of the close genetic relationships among the 150 jujube accessions.

### Estimation of Linkage Disequilibrium

**FIGURE 7:**
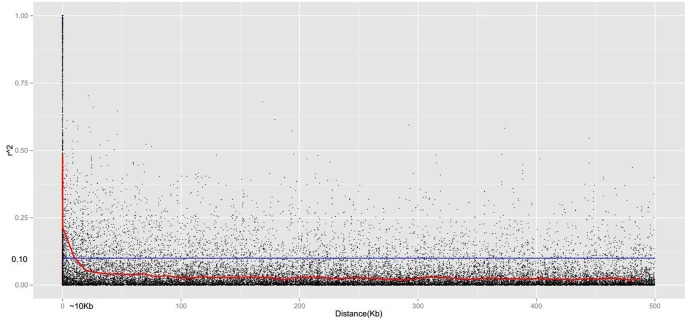
**Linkage disequilibrium decay for all pseudo-chromosomes**. Pairwise correlations between 4,680 SNPs are plotted against the physical distance (Kb) between in base pairs. The *curves* indicate the non-linear regressions of *r*^2^ onto the physical distance in base pairs. The *black* plot shows the density distribution of *r*^2^ values and the *blue* line indicates the derived threshold for linkage disequilibrium (LD) due to linkage.

Linkage disequilibrium is an important consideration for marker-assisted selective breeding and genome-wide association studies. However, there are currently no reports regarding LD in Chinese jujube. In this study, the distributions of *r*^2^ associated with physical distance for all pseudo-chromosomes and individual pseudo-chromosomes were determined based on genotyping information for 4,680 genome-wide SNPs in 150 Chinese jujube accessions. A rapid decline was observed with increasing physical distance. The decrease was uniform for all pseudo-chromosomes (**Figure 7**), but not for individual pseudo-chromosomes (data not shown). All of the LD decays (i.e., *r*^2^ < 0.1) were estimated to a physical distance of 10 kb (Supplementary Tables S6, S7). For all pseudo-chromosomes, the estimated LD was very low, with few instances of *r*^2^ > 0.5 (**Figure 7**).

## Discussion

Genotyping-By-Sequencing has many key advantages including low cost, reduced sample handling, few purification steps, no size fractionation, efficient barcoding and easiness to scale up (Davey et al., 2011; Elshire et al., 2011). These advantages make GBS an ideal method for investigating genomic diversity (Lam et al., 2010; Lu et al., 2013; Wong et al., 2015), constructing genetic linkage maps (Ma et al., 2012; Poland et al., 2012; Liu H. et al., 2014), and conducting genome-wide association studies (Singh et al., 2013; Uitdewilligen et al., 2013; Lin et al., 2015) in plants.

### Genome-wide SNPs Discovery and Genotype Using a GBS Assay

The maximum and minimum numbers of clean reads were obtained for BJFU-66 and BJFU-435, respectively (Supplementary Table S2). Although there was an insufficient number of clean reads for BJFU-435, 23 SNPs distributed throughout the genome were detected which were effective to achieve our study objective. The variation in the number of reads recovered in GBS studies may be due to restriction enzyme site variations and differences in methylation (Pan et al., 2015). The number of high-quality SNPs identified in a GBS experiment may be affected by genome size, the sequencing depth, and the study objectives. For instance, to construct a saturated genetic linkage map and to identify a known quantitative trait locus related to apple skin color, 81 individuals from an F_1_ population segregating for skin color were studied using GBS (6-fold sequencing depth). A total of 3,967 SNPs were finally identified (Gardner et al., 2014), which is fewer than the number of SNPs detected in this study. In a previous chickpea investigation, 93 diverse desi chickpea cultivars were analyzed using GBS (∼30-fold sequencing depth) to extrapolate the natural allelic diversity and domestication patterns. Researchers identified 20,439 and 24,405 high-quality SNPs in the desi and kabuli chickpea genomes, respectively (Kujur et al., 2015), which were both higher than the number of SNPs detected in this study. During a jujube study, Zhang et al. (2016) combined three restriction enzymes (i.e., *Mse*I, *Hae*III, and *EcoR*I) when preparing a GBS library. They ultimately identified 2,540 high-quality SNP markers, which were used to construct an integrated genetic linkage map. Zhang et al. (2016) developed fewer SNP markers than we did in our study, which involved a digestion step with *ApeK*I. Additionally, we identified tri-allelic (0.80%) SNPs. Definitely, reducing DNA treatments and applying stricter filter conditions during a GBS assay will decrease the number of multi-allelic SNPs. Thirty-eight tri-allelic SNPs were identified using the default parameters of the GATK program. We detected a single position for each tri-allelic SNP, which confirmed their validity (Supplementary Table S3). The cost of next-generation sequencing platforms is much lower than that of the Sanger sequencing technique (Frank et al., 2013). Additionally, although the cost per polymorphic locus is similar for GBS and genotyping-in-thousands by sequencing (i.e., another next-generation sequencing approach) (Campbell et al., 2015), GBS can detect nearly 25-fold more polymorphic loci. This indicates that GBS is an efficient and cost-effective genotyping approach (Pértille et al., 2016). The sensitivity of the GBS technique to methylation enables more extensive cutting in single-copy gene-rich genomic regions (Sonah et al., 2013). Previous studies revealed that the distribution of SNP markers is skewed in favor of gene-rich regions (Mayer et al., 2012; Sonah et al., 2013) as well as centromeric and pericentromeric regions (Poland et al., 2012). The genomic distribution of SNPs is not homogenous, and regional differences in recombination rates may be responsible for a substantial proportion of the variability in nucleotide polymorphism levels (Nachman, 2001). The existence of mutation hotspots is another possible reason for the fluctuations in SNP density (Rogozin and Pavlov, 2003). Furthermore, chromosomes may be affected by various selection pressures that influence gene density (Barreiro et al., 2008). In the jujube genome, the density of genes, repeats, and SSRs was specific to each chromosome (Liu M. et al., 2014). Thus, varying SNP frequencies on different pseudo-chromosomes of approximately the same size were observed. Consistent with previous studies involving jujube (Zhao et al., 2014), chickpea (Agarwal et al., 2012; Jain et al., 2013), and rice (Parida et al., 2012), we observed that transitions were more frequent than transversions.

### Genetic Diversity, Population Structure, and Linkage Disequilibrium of the 150 Chinese Jujube Accessions

According to Botstein et al. (1980), the PIC value is equal or greater than 0.5 which suggested high informative with a SSR marker loci. The PIC values for bi-allelic SNP markers range from 0 to 0.5, whereas for multi-allelic SSR markers, the PIC value can be as high as 0.5–1.0. Therefore, our calculated PIC values for SSRs (0.59), tri-allelic (0.38), and bi-allelic (0.27) SNPs implying that the 150 Chinese jujube accessions were highly genetically diverse. Based on the sharp peak for the delta-K value and the results of the PCoA, the 150 jujube accessions were classified into two groups. However, in our previous studies, 962 Chinese jujube accessions (including the 150 Chinese jujube accessions analyzed in this study) were grouped into three clusters based on the delta-K value and PCoA related to 24 SSRs (Xu et al., 2016). This discrepancy may be due to the magnitudes of the Chinese jujube accessions. Similar results were observed in rice, where the population was classified into three groups by Zhao et al. (2009) while it was classified into seven groups by Jin et al. (2010), more groups with the latter is primarily due to the higher number and diverse set of germplasm (Jin et al., 2010).

The distance over which LD persists determines the number and density of markers. It also clarifies the experimental design needed for an association analysis. Our Chinese jujube study revealed a relatively rapid LD decay within a short range (i.e., ∼10 kb) for all pseudo-chromosomes and individual pseudo-chromosomes. The LD level in plants can be affected by reproductive systems (Arunyawat et al., 2012). The LD decay estimated in this study was lower than that of sorghum (Morris et al., 2013) and rice (Mather et al., 2007), which are self-pollinated species. These observations were consistent with the expected results. LD decays more rapidly in cross-pollinated species than in self-pollinated species because recombination is less effective in the latter species type (Flint-Garcia et al., 2003). In cross-pollinated species, the LD level can be affected by population size as well as by domestication and breeding during evolution. The extent of the LD decay for all Chinese jujube pseudo-chromosomes was lower than that of other fruit tree species, such as sweet cherry (Campoy et al., 2016) and apple (Kumar et al., 2012), while it was higher than that of grape (Lijavetzky et al., 2007). These results are likely because of the effects of genetic drift, which can lead to the loss of rare allelic combinations in small populations (Flint-Garcia et al., 2003). The low *r*^2^ values observed in this study may have been affected by the use of markers with low genome coverage, similar to what was observed in a sorghum study (Morris et al., 2013). Low values of *r*^2^ in this study may be also affected by using low genome coverage of markers. These results may serve as an important foundation for future applications of genome-wide association studies and marker-assisted selective breeding of Chinese jujube.

### Comparison of SSR and SNP Markers Related to Genetic Diversity and Population Structure

Different mutational processes govern allelic variations at SSR and SNP loci, with lower mutation rates for SNPs than for SSRs (Guichoux et al., 2011). Additionally, differences in generated mechanisms (i.e., replication slippage for SSRs vs. point mutations for SNPs) influence the variability in marker utility during diverse analyses (Singh et al., 2013).

In terms of genetic diversity, the highest PIC value among the three data sets was associated with the SSRs. The PIC values based on SSRs and bi-allelic SNPs were higher in this study than in a previous study involving rice (Singh et al., 2013), while they were lower than the values for maize (Yang et al., 2011). The PIC value is likely influenced by many factors, such as the breeding behavior of the species, genetic diversity in the collection, size of the collection, sensitivity of the genotyping method, and the genomic locations of markers. Single nucleotide polymorphism markers with relatively low mean PIC values may be more informative than SSR markers with high mean values (Singh et al., 2013). Therefore, the average PIC value (0.59) in this study, which was calculated based on the SSRs, tri-allelic SNPs (0.38), and bi-allelic SNPs (0.27), indicates that the two types of markers exhibit a similar resolving power.

According to theoretical expectations, the distribution of allele frequencies differed between the SNP and SSR markers. The SSR loci were more common in the population at low frequencies, whereas the tri-allelic and bi-allelic SNP loci were more abundant at intermediate frequencies in this study. These results are consistent with those of previous studies (Laval et al., 2002; Vignal et al., 2002). SSRs are often dominated by rare alleles, while SNPs with a MAF < 0.05 were discarded in this GBS-SNP study, which may be the two important considerations to explain it.

The cost per polymorphic locus for the SSR procedure was higher than that for the GBS-SNP procedure. However, fewer SSR markers were required for examining the genetic diversity and population structure of the jujube core collection. Thus, the total cost of the SSR procedure was much lower than the corresponding cost for the GBS-SNP procedure. The cost per polymorphic locus for the SSR procedure was higher than that for the GBS-SNP procedure. However, fewer SSR markers were required for examining the genetic diversity and population structure of the jujube core collection. Thus, the total cost of the SSR procedure was much lower than the corresponding cost for the GBS-SNP procedure.

In terms of population structure, based on the analysis of PCoA and neighbor-joining trees for the two marker types, the 150 Chinese jujube accessions were grouped into same numbers of clusters with similar broad patterns, however, the number of accessions grouped into the clusters differed. These findings were not surprising because a broad grouping pattern is expected regardless of the marker types used in investigations of genetic relatedness. Similar findings were reported in rice (Courtois et al., 2012; Singh et al., 2013). The low percentage of admixture accessions revealed by the STRUCTURE analyses based on the two marker types suggests that there were relatively few domestication or breeding events during evolution since admixture is the representation of diverse parents. Although cluster differentiations were apparent, there was no geographical isolation for the analyzed Chinese jujube accessions, possibly because of frequent transfers of accessions between the two sites (Cangzhou and Taigu). The percentage of admixture accessions determined during STRUCTURE analyses and the proportion of cumulative variation in PCoA analysis were higher for the SSR markers than for the SNP markers. These results indicate that the resolving power for population structure was higher for SSR markers than for SNP markers. This conclusion is consistent with the findings of previous studies (Hamblin et al., 2007; Li et al., 2010; Yang et al., 2011). Li et al. (2010) explained that the disparity between the two markers is related to the number of observed alleles. The resolving power for clustering increases as the number of observed alleles increases. However, in this study, the SSR markers exhibited better clustering power, with 209 observed alleles for the SSRs compared with 9,398 observed alleles for the SNPs.

Although the number of clusters differed among the PCoA, STRUCTURE, and neighbor-joining tree analyses, the same clusters were obtained using SNP- and SSR-based methods for each analysis. This indicted the clusters generated in this study for the 150 accessions were real. Similar results were reported by Li et al. (2010) and Singh et al. (2013).

Several studies concluded that many SNPs are required to obtain the same information as SSR markers (Laval et al., 2002; Yu et al., 2009; Van Inghelandt et al., 2010). In this study, although there were nearly 195-times more bi-allelic SNPs than SSRs, the two types of markers performed inconsistently during analyses of genetic diversity and population structure. Thus, our findings suggest the resolving power of the two marker types is unrelated to the number of SNPs and observed alleles. Instead, it is associated with the characteristics of the markers and the studied species.

## Conclusion

Genome-wide SNPs for diverse jujube germplasm were identified in this study. They were subsequently applied to assess the genetic diversity, population structure, and LD of Chinese jujube accessions. This is the first report describing the LD pattern in Chinese jujube. We revealed that GBS technology is a powerful tool for identifying and genotyping SNPs at a genome-wide scale. The PIC values calculated based on the SSRs and SNPs suggest the 150 Chinese jujube accessions are highly genetically diverse. The two markers exhibited similar resolving power regarding genetic diversity. However, the resolving power of the SSRs was higher than that of the SNPs in terms of population structure. Our findings may help researchers select suitable SSRs and/or SNPs for various analyses of Chinese jujube. They may also serve as a useful source of genetic information relevant for future genome-wide association studies and/or marker-assisted selective breeding.

## Author Contributions

YL designed the conception and experiment; WC performed the experiments; WC and LH collected and analyzed the data; WC wrote the manuscript; XP and LH provided valuable suggestions on the manuscript; ZZ, LH, XP, and YL revised the manuscript; YL obtained funding and is responsible for this article. All authors read and approved the manuscript.

## Conflict of Interest Statement

The authors declare that the research was conducted in the absence of any commercial or financial relationships that could be construed as a potential conflict of interest.

## Footnotes

1 https://github.com/BGI-shenzhen/PopLDdecay
